# The role of prescribed opioids in Norwegian overdose deaths in 2005–2021

**DOI:** 10.1007/s00228-025-03897-5

**Published:** 2025-08-11

**Authors:** Hilde Marie Erøy Edvardsen, Thomas Clausen, Stig Tore Bogstrand, Svetlana Skurtveit

**Affiliations:** 1 Section of Forensic Research, Oslo University Hospital, Oslo, Norway; 2 Norwegian Centre for Addiction Research (SERAF), University of Oslo, Oslo, Norway; 3 Department of Public Health Science, University of Oslo, Oslo, Norway; 4 Department of Chronic Diseases, Norwegian Institute of Public Health, Oslo, Norway

**Keywords:** Pharmaceutical opioids, Prescription rate, Overdose death, Poly-drug use, Benzodiazepines, Psychoactive drugs

## Abstract

**Purpose:**

Pharmaceutical opioids significantly contribute to overdose deaths in Norway. This study investigated the prevalence of prescriptions and examined characteristics of those with filled prescriptions, comparing them to individuals without prescriptions.

**Methods:**

This cohort study encompassed overdose deaths from 1.1.2005 to 31.12.2021, using toxicology and data linkage from nationwide health registries. Approximately 90% of Norwegian forensic autopsy cases were linked to the Norwegian Cause of Death Registry and the Norwegian Prescribed Drug Registry. The study included cases with overdose as cause of death, as defined by the European Union Drugs Agency (EUDA), along with benzodiazepine- and GHB-induced deaths (*N* = 3764). Detection and filled prescription of oxycodone, codeine, buprenorphine, methadone, tramadol, and fentanyl were studied.

**Results:**

Fentanyl had the lowest proportion of filled prescriptions prior to death (27%), whereas codeine had the highest (67%). Buprenorphine and methadone, primary medications used in opioid agonist treatment (OAT) in Norway, showed low rates of filled prescriptions (33% and 29%, respectively). Women and older adults exhibited a higher prevalence of filled prescriptions compared to men and younger individuals. More than half of the individuals with prescribed opioids also had benzodiazepines prescribed and/or in their blood prior to death.

**Conclusion:**

A substantial proportion of the deceased had filled prescriptions for opioids, indicating that medical treatment might be a crucial source for these substances in overdose deaths. Additionally, poly-drug use was common, especially opioids and benzodiazepines, highlighting the need for prescribers to be cautious.

## Introduction

In Norway, there are approximately 290 annual overdose deaths, reflecting a rate of 7.1/100.000 inhabitants in 2023 [1]. This figure has shown an increasing trend over the past decade, highlighting the importance of a better knowledgebase for improved targeted preventive measures. Since 2014, the Norwegian Directorate of Health has established a national overdose prevention strategy that includes several interventions [2]. Most of these recommendations have primarily focused on heroin-related deaths, which have notably decreased compared to two decades ago. However, there is a concerning trend where overdose deaths involving pharmaceutical opioids are increasing [3, 4].

This shift is partly due to other populations experiencing overdoses from opioids [5]. Over the past decade, Norway has undergone a change of the prescription scheme for medical opioids towards a more liberal scheme, particularly pertaining to the treatment of chronic pain [5]. In 2008, these medications became eligible for reimbursement through the National Insurance Scheme when prescribed for chronic pain by designated specialist physicians [6]. The National Insurance Scheme is publicly funded and overseen by the Norwegian Health Economics Administration [7]. In 2016, the prescription scheme was further extended to include prescriptions for chronic pain issued by general practitioners, allowing for reimbursement under the same scheme [6]. A liberalization is also seen internationally, with a particular crisis in the USA [8]. Countries like the UK, Ireland, and Northern Ireland have reported increases in opioid-related issues [9]. Some regions are seeing rising prescription rates, which raises alarms about potential future crises.

The demographic profile of individuals who die from overdose due to pharmaceutical opioids tends to differ from that of traditional heroin overdose cases in Norway. Specifically, these individuals are typically older, often female, and frequently have chronic pain conditions or a history of cancer [10]. To effectively mitigate these overdose deaths, it is crucial to investigate whether the pharmaceutical medicines involved were obtained through prescriptions. Given that prescribed opioids are part of the problem, targeted interventions should be directed towards both prescribers and patients to enhance safety and reduce the risk of overdose.

Oxycodone, codeine, buprenorphine, methadone, tramadol, and fentanyl are the most frequently detected opioids in autopsy cases in Norway, without having challenging toxicological interpretational issues, in contrast to morphine [11]. Drug-induced deaths rarely constitute mono-intoxications in Norway, and benzodiazepines is the most prevalent drug group, which is co-detected in such deaths [3, 4]. The increased death risk of this drug combination is well known, so addressing whether the combination use comes from prescriptions or not will fill the knowledge gap regarding where typical used drugs were obtained before a lethal overdose. Registry-based studies, which have been conducted in Norway previously [5, 10], cannot explore these hypotheses since the actual drug intake is not addressed when toxicological data is missing.

This study presents a unique dataset that integrates toxicological results from autopsies with prescription data and the recorded cause of death.

### Aims

The purpose of this study was to determine whether victims from overdose deaths involving the most prevalent, and potentially prescribed, pharmaceutical opioids had filled prescriptions for these specific drugs within the 2 months preceding death. To better understand the variation in sub-group characteristics, we further aimed to examine the characteristics of individuals who died with a filled prescription of the substance later detected in autopsy, in comparison to those without prescription and to explore multivariate predictors including age, sex, poly-drug use, benzodiazepine consumption, illicit (non-pharmaceutical) drug use, temporal trends, and instances of suicide.

## Methods

### Study design

This is a cohort study of deceased overdose victims with record linkages. Autopsy cases from the Department of Forensic Sciences at Oslo University Hospital were linked to the CoDR, as detailed by Jamt et al. [12] and Edvardsen and Clausen [3]. Included cases were those classified as drug-induced deaths according to the European Union Drugs Agency (EUDA) definition, based on ICD-10 codes (*n* = 3,544), known as “EMCDDA definition for the general mortality registers, formerly known as Selection B.” The definition states that cases are counted when the underlying cause of death is attributed to mental and behavioral disorders resulting from psychoactive substance use or poisoning [13]. In addition, we included deaths induced by benzodiazepines and GHB (*n* = 220) since they are not covered by the EUDA definition, and we regarded them as relevant due to their sedative and toxic properties.

### Data sources

#### Forensic toxicology

As detailed by Jamt et al., toxicological analyses were per standard routine conducted on approximately 900 substances, including recreational drugs, medicinal products, metabolites, and toxins present in the blood [12].

#### Pharmacological considerations and drug groups

Edvardsen and Clausen previously described the methodological considerations for interpreting toxicological results prior to data analysis [3]. These are used in the present study, but for codeine, we employed a detection limit set at 150 ng/mL. Concentrations of codeine below this threshold were attributed to heroin intake and were therefore excluded from calculations regarding codeine use.

The pharmaceutical opioids included in this study represent the most detected opioids in Norwegian overdose deaths, as identified by Edvardsen and Clausen [3]. These opioids are oxycodone, codeine, buprenorphine, methadone, tramadol, and fentanyl.

The association variable for co-detection of benzodiazepines and/or Z-drugs “BZDs and Z-drugs” encompasses the following substances: Alprazolam, diazepam, N-desmethyldiazepam, clonazepam, 7-aminoclonazepam, midazolam, nitrazepam, 7-aminonitrazepam, oxazepam, zolpidem, and zopiclone. These drugs are marketed in Norway and may be prescribed by healthcare professionals.

The association variable “non-pharmaceutical drugs” includes detection of at least one of a wide range of substances, such as 1-benzylpiperazine, 2,4-dinitrophenol, 2CB, 2-phenylethylamine, 2-fluoromethamphetamine, 3-methylmethcathinone, 3,5-dimethoxyphenol, 4-HO-EPT, 4-fluoroamphetamine, 4-fluoromethamphetamine, 4-methylamphetamine, 5-APB, 5F-APINACA, 5-OH-DMT, 5-MeO-DMT, 8-aminoclonazolam, AH-7921, acetylfentanyl, alpha-methyltryptamine, alpha-PVP, alfentanil, amphetamine, APINACA, cocaine/benzoylecgonine, bromazepam, bromo-dragonFLY, cyclopropyl fentanyl, despropionyl 4-F-fentanyl, diclazepam, estazolam, etizolam, phenazepam, flualprazolam, flubromazolam, flurazepam, furanylfentanyl, homoamphetamine, carfentanil, lorazepam, LSD, mCPP, MDA, MDMA, MDPV, methamphetamine, methoxetamine, methoxyacetylfentanyl, mitragynine, heroin, piritramide, PMA, PMMA, psilocybin, remifentanil, TFMPP, THC, tryptamine, and GHB. Some of these drugs, lorazepam, flurazepam, etizolam, and estazolam, are not marketed in Norway and therefore considered non-pharmaceutical.

#### Cause of death

Norway’s annual overdose death statistics use diagnostic codes from the Norwegian Cause of Death Registry (CoDR), based on WHO’s ICD-10 [1]. The cause of death is determined with manner of death. The manner of death indicated whether it was homicide, suicide, accidental, or with undetermined intent. Overdose death codes are generally not substance-specific, except for methadone and heroin. Data are grouped into categories like “other opioids” (codeine, morphine, oxycodone) and “other synthetic narcotics” (buprenorphine, tramadol, fentanyl). CoDR statistics do not allow analysis of individual substances, and prescription database studies cannot confirm if opioids were ingested or detected at death. Registry-based studies that utilize a prescription database only are unable to ascertain whether pharmaceutical opioids were ingested or detected at the time of death.

#### Prescription data

The cases were linked to the Norwegian Prescribed Drug Registry (NorPD) for information on filled opioid prescriptions. NorPD includes data from all pharmacies in Norway, categorized by WHO’s Anatomical Therapeutic Chemical (ATC) classification system, including dispensing dates [14]. We focused on prescriptions filled within 2 months before death. The opioids analyzed were oxycodone (N02AA05 and N02AA55), fentanyl (N02AB03), buprenorphine (N02AE01, N07BC01, and N07NC51), codeine (N02AJ06), tramadol (N02AJ13 and N02AX02), and methadone (N07BC02 and N07BC05), detected at autopsy. For drugs with multiple ATC codes, individual products were not differentiated.

Regarding “Dispensed BZDs/Z-drugs,” the study included alprazolam (N05BA12), diazepam (N05BA01), clonazepam (N03AE01), midazolam (N05CD08), nitrazepam (N05CD02), oxazepam (N05BA04), zolpidem (N05CF02), and zopiclone (N05CF01).

Prescription data is inferred from the detection of individual opioids at autopsy. For instance, when results regarding oxycodone is reported, it indicates that oxycodone was detected during the autopsy.

Morphine data was excluded due to its complex toxicological interpretation, but morphine-positive cases were included in the study. Morphine is often detected as a metabolite of substances like heroin and codeine, complicating its analysis.

#### Cancer diagnosis

We used data from the Norwegian Patient Registry to determine if the deceased had received a cancer diagnosis (ICD-10 codes C00-C97, malignant neoplasms). The registry had data available for the period 2008–2021.

### Statistical analysis

Normality of the continuous variables age and number of detected substances was assessed using the Shapiro–Wilk test. Bi-variate associations were analyzed using Pearson’s chi-square test for categorical variables (sex assigned at birth), manner of death as suicide, prescription of benzodiazepines, co-detection of benzodiazepines, and co-detection of at least one non-pharmaceutical drug. Mean age and difference in the number of psychoactive drugs detected were compared using Student’s *T*-test. A significance level was established at *p* < 0.05. Multivariate logistic regression was used to calculate odds ratios (ORs) and adjusted odds ratios (aORs), with variables included based on theoretical relevance and a significance level of *p* < 0.25 in the bi-variate analysis. Temporal trends were included in the logistic regression as a continuous variable. A significance level was established at *p* < 0.05. All analyses were performed using the Statistical Package for the Social Sciences (SPSS Statistics), version 29, Armonk, New York: IBM Corp.

### Ethics approval declaration

The study was approved by the Regional Ethical Committee South-East Section D (Ref No. 255254) and by the Public Prosecutor at the Office of the Director of Public Prosecutions (22/1933). The project is registered at the Data Protection Officer at Oslo University Hospital (21/10388). In Norway, privacy in registry-based research is maintained through several measures: de-identification of data, encryption to prevent unauthorized access, approval by ethical committees, and secure storage of sensitive data. In this specific project, only age, month/year of death, and month/year of prescription dispensation are provided, further ensuring privacy and making re-identification difficult. These measures collectively ensure ethical research practices.

## Results

### Sample characteristics

From 2005 to 2021, 3764 overdose deaths were analyzed, with 73% being male and 16% classified as suicides (see Table 1). When the individual opioids were detected, 67% had filled prescriptions for codeine, 58% for tramadol, 53% for oxycodone, 33% for buprenorphine, 29% for methadone, and 27% for fentanyl within the 2 months before death (see Table 1). This did not differ significantly from prescriptions filled in the year preceding death (results not shown). Overall, 36% had filled a prescription for at least one opioid. Combining codeine, oxycodone, and tramadol showed a 60% prevalence, while buprenorphine, methadone, and fentanyl showed 29%. Benzodiazepine prescriptions were present in 50% of cases with benzodiazepines detected at death.

**Table 1:** Sample characteristics with prescription and demographic data for the individual opioids detected. The analysis compares individuals with and without filled prescriptions for the opioid also detected at autopsy

Detected opioid (*n*)		All (*N* = 3764)	Women* (*n* = 1019)	Men* (*n* = 2745)	Age, 95% CI**	Suicide*
Oxycodone (*n* = 316)	With prescription	169 (53%)	86 (65%)	83 (45%)	49.9 (47.9–51.9)	62 (62%)
	Without prescription	147 (47%)	46 (35%)	101 (55%)	41.9 (39.3–44.5)	38 (38%)
	*P*-value		*P* < 0.001		*P* < 0.001	*P* = 0.039
Codeine (*n* = 326)	With prescription	217 (67%)	115 (73%)	102 (61%)	51.5 (49.9–53.0)	85 (59%)
	Without prescription	109 (33%)	43 (27%)	66 (39%)	47.6 (45.0–50.2)	58 (41%)
	*P*-value		*P* = 0.021		*P* = 0.009	*P* = 0.016
Buprenorphine (*n* = 228)	With prescription	76 (33%)	26 (44%)	50 (30%)	45.3 (43.1–47.5)	6 (27%)
	Without prescription	152 (67%)	33 (56%)	119 (70%)	38.3 (36.1–40.5)	16 (73%)
	*P*-value		*P* = 0.042		*P* < 0.001	*P* = 0.526
Methadone (*n* = 798)	With prescription	229 (29%)	65 (36%)	165 (27%)	47.4 (46.2–48.6)	21 (31%)
	Without prescription	569 (71%)	115 (64%)	453 (73%)	38.4 (37.5–39.4)	46 (69%)
			*P* = 0.014		*P* < 0.001	*P* = 0.634
Tramadol (*n* = 220)	With prescription	128 (58%)	66 (64%)	62 (53%)	50.4 (48.1–52.6)	54 (64%)
	Without prescription	92 (42%)	38 (36%)	54 (47%)	41.0 (37.9–44.1)	30 (36%)
	*P*-value		*P* = 0.133		*P* < 0.001	*P* = 0.149
Fentanyl (*n* = 180)	With prescription	48 (27%)	24 (38%)	24 (21%)	49.5 (45.5–53.5)	11 (38%)
	Without prescription	132 (73%)	39 (62%)	93 (79%)	39.3 (37.0–41.5)	18 (62%)
	*P*-value		*P* = 0.011		*P* < 0.001	*P* = 0.134

*Chi-square test; **Independent samples *T*-test

In total, 4.1% of the deceased had a cancer diagnosis post-2008. Among those who had at least one opioid prescription in the last year before death, 7.0% had a cancer diagnosis.

### Differences in prevalence of filled prescriptions

Table 1 shows that women had a significantly higher prevalence of filled prescriptions for oxycodone and codeine (*p* < 0.001). In contrast, men had a higher prevalence of filled prescriptions for codeine (*p* < 0.001). Additionally, the proportion of men without filled prescriptions was higher for oxycodone (*p* < 0.001), buprenorphine (*p* < 0.05), methadone (*p* < 0.05), and fentanyl (*p* < 0.05). Conversely, women were significantly more likely to be without filled prescriptions for buprenorphine, methadone, and fentanyl (*p* < 0.05).

The mean age was significantly higher for all studied opioids in the prescription groups compared to the non-prescription groups. The age differences ranged from 3.9 years for codeine to 10.2 years for fentanyl (see Table 1).

The number of psychoactive drugs detected was significantly higher among those with filled prescriptions for tramadol, compared to those without prescription of tramadol (4.77 vs. 4.36, *p* < 0.001) and among those without filled prescriptions for methadone (4.18 vs. 3.90, *p* = 0.042).

Individuals with at least one BZD/Z-drug prescription and/or BZDs/Z-drugs detected were significantly more likely to have filled prescriptions for oxycodone, codeine, and tramadol (see Table 2). Conversely, individuals with at least one BZD/Z-drug prescription were significantly more likely to be missing filled prescriptions for buprenorphine, methadone, and fentanyl.

**Table 2:** Prescription and other drugs data for the individual opioids detected. The analysis compares individuals with and without filled prescriptions for the same opioid detected at autopsy

		Number of psychoactive drugs (mean, 95% CI)**	Co-prescription of benzodiazepines*	Co-detection of benzodiazepines*	Co-detection of non-pharmaceutical drugs*
Oxycodone	With prescription	4.35 (4.09–4.61)	143 (66%)	135 (56%)	30 (29%)
	Without prescription	4.32 (4.00–4.64)	74 (34%)	106 (44%)	74 (71%)
	*P*-value	*P* = 0.887	*P* < 0.001	*P* = 0.105	*P* < 0.001
Codeine	With prescription	4.50 (4.28–4.73)	195 (77%)	189 (70%)	30 (48%)
	Without prescription	4.20 (3.85–4.55)	57 (23%)	82 (30%)	32 (52%)
	*P*-value	*P* = 0.143	*P* < 0.001	*P* = 0.007	*P* < 0.001
Buprenorphine	With prescription	4.58 (4.10–5.05)	44 (40%)	63 (33%)	46 (30%)
	Without prescription	4.24 (3.98–4.49)	65 (60%)	130 (67%)	107 (70%)
	*P*-value	*P* = 0.168	*P* = 0.031	*P* = 0.603	*P* = 0.135
Methadone	With prescription	3.90 (3.68–4.13)	126 (33%)	167 (26%)	139 (27%)
	Without prescription	4.18 (4.04–4.32)	262 (67%)	471 (74%)	370 (73%)
	*P*-value	*P* = 0.042	*P* = 0.027	*P* < 0.001	*P* = 0.210
Tramadol	With prescription	4.77 (4.38–5.17)	92 (65%)	87 (55%)	40 (49%)
	Without prescription	4.36 (4.03–4.69)	49 (35%)	71 (45%)	41 (51%)
	*P*-value	*P* < 0.001	*P* = 0.005	*P* = 0.134	*P* = 0.043
Fentanyl	With prescription	4.44 (3.91–4.97)	36 (37%)	33 (26%)	11 (13%)
	Without prescription	4.12 (3.83–4.41)	61 (63%)	95 (74%)	72 (87%)
	*P*-value	*P* = 0.277	*P* < 0.001	*P* = 0.673	*P* < 0.001

*Chi-square test; **Independent samples *T*-test

The prevalence of filled opioid prescriptions was significantly lower among deceased individuals who had non-pharmaceutical drugs detected at autopsy. This was observed for several opioids: oxycodone, codeine, tramadol, and fentanyl (see Table 2).

### Multivariate associations with filled prescriptions

Table 3 presents univariate and multivariate analyses, with focus on adjusted associations between various factors and the likelihood of having filled opioid prescriptions. Women were more likely than men to have filled prescriptions for oxycodone (aOR 1.77, 95% CI 1.07–2.93), codeine (aOR 1.77, 95% CI 1.05–2.99), and methadone (aOR 1.53, 95% CI 1.04–2.25).

**Table 3:** Factors associated with filled opioid prescriptions in the last 2 months. The analysis compares individuals with and without filled prescriptions for the same opioid detected at autopsy (results are based on detection and prescription data). Odds ratios (ORs) are from univariate analyses, while adjusted odds ratios (aORs) are from multivariate analyses including age, sex, and variables with *P* < 0.25 in univariate analyses. Adjusted odds ratios are presented with 95% confidence intervals (95% CIs)

		Women (reference men)	Age, 95% CI	Number of psychoactive drugs (mean, 95% CI)	Concomitant findings of benzodiazepines	Concomitant findings of non-pharmaceutical drugs	Trend with time	Suicide
Oxycodone	OR (95% CI)	2.28 (1.44–3.61)*	1.04 (1.02–1.06)*	1.01 (0.89–1.14)	1.54 (0.91–2.59)	0.21 (0.13–0.36)*	1.01 (0.96–1.07)	1.66 (1.02–2.70)**
	aOR (95% CI)	1.77 (1.07–2.93)**	1.02 (1.01–1.04)**		1.67 (0.95–2.95)	0.27 (0.16–0.47)*		0.98 (0.57–1.70)
Codeine	OR (95% CI)	1.73 (1.08–2.76)**	1.03 (1.01–1.05)**	1.11 (0.97–1.27)	2.22 (1.23–4.00)**	0.39 (0.22–0.68)*	0.98 (0.94–1.03)	0.57 (0.36–0.90)**
	aOR (95% CI)	1.77 (1.05–2.99)**	1.02 (1.00–1.04)	1.08 (0.92–1.28)	2.13 (1.09–4.16)**	0.34 (0.17–0.65)*		0.41 (0.24–0.69)*
Buprenorphine	OR (95% CI)	1.86 (1.02–3.45)**	1.05 (1.02–1.07)*	1.12 (0.96–1.30)	0.82 (0.39–1.73)	0.65 (0.36–1.15)	1.05 (0.98–1.12)	0.73 (0.27–1.94)
	aOR (95% CI)	1.39 (0.71–2.71)	1.04 (1.02–1.07)*	1.16 (0.97–1.40)		0.66 (0.34–1.27)	1.01 (0.94–1.09)	
Methadone	OR (95% CI)	1.55 (1.09–2.21)**	1.08 (1.06–1.10)*	0.91 (0.83–0.997)**	0.55 (0.38–0.79)*	0.82 (0.60–1.12)	1.08 (1.05–1.12)*	1.14 (0.66–1.96)
	aOR (95% CI)	1.53 (1.04–2.25)**	1.08 (1.06–1.10)*	1.08 (0.95–1.22)	0.43 (0.27–0.70)*	1.07 (0.74–1.56)	1.05 (1.01–1.099)**	
Tramadol	OR (95% CI)	1.51 (0.88–2.60)	1.05 (1.03–1.07)*	0.89 (0.78–1.03)	0.63 (0.34–1.16)	0.57 (0.32–0.99)**	1.01 (0.95–1.08)	1.51 (0.86–2.64)
	aOR (95% CI)	1.22 (0.67–2.23)	1.05 (1.03–1.07)*	0.96 (0.80–1.14)	0.65 (0.32–1.33)	1.09 (0.56–2.13)		1.35 (0.72–2.53)
Fentanyl	OR (95% CI)	2.39 (1.21–4.70)**	1.06 (1.03–1.08)*	1.11 (0.92–1.34)	0.86 (0.42–1.76)	0.25 (0.12–0.53)*	1.07 (0.99–1.15)	1.88 (0.82–4.35)
	aOR (95% CI)	1.63 (0.77–3.45)	1.05 (1.02–1.07)**			0.29 (0.13–0.65)**	1.08 (0.99–1.18)	0.92 (0.36–2.31)

*P < 0.001; **P < 0.05

Age differences appeared as older age was generally associated with a higher likelihood of having filled prescriptions for several opioids. Specifically, the odds ratios were: oxycodone (aOR 1.02, 95% CI 1.01–1.04), buprenorphine (aOR 1.04, 95% CI 1.02–1.07), methadone (aOR 1.08, 95% CI 1.06–1.10), and fentanyl (aOR 1.05, 95% CI 1.02–1.07).

The concomitant detection of at least one BZD/Z-drug was significantly associated with having filled prescriptions for codeine (aOR 2.13, 95% CI 1.09–4.16). Conversely, the co-detection of BZDs/Z-drugs was associated with lower odds of having filled prescriptions for methadone (aOR 0.43, 95% CI 0.27–0.70).

The co-detection of at least one non-pharmaceutical drug was significantly associated with lower odds of having filled prescriptions for oxycodone (aOR 0.27, 95% CI 0.16–0.47), codeine (aOR 0.34, 95% CI 0.17–0.65), and fentanyl (aOR 0.29, 95% CI 0.13–0.65).

The proportion of filled prescriptions for methadone increased significantly from 18% in 2005–2009 to 36% in 2018–2021, while the other opioids did not show significant changes in the prescription rate (aOR 1.05, 95% CI 1.01–1.099, *p* < 0.05) (see Table 3).

## Discussion

This study, which encompasses most Norwegian overdose deaths over a 17-year period, represents the first comprehensive investigation in Norway, and one of few studies, examining filled prescriptions within the 2 months before death for various opioids detected at autopsy. Combining codeine, oxycodone, and tramadol showed a 60% prevalence of filled prescriptions. Fentanyl had the lowest proportion of filled prescriptions prior to death, while codeine exhibited the highest proportion. Buprenorphine and methadone, which are the two most used medications in opioid agonist treatment (OAT) in Norway [15], also demonstrated a relatively low share of filled prescriptions; less than one-third of the deceased with these substances present in their blood had filled a prescription within the 2 months preceding death. Additionally, women and older individuals generally exhibited a higher prevalence of filled prescriptions compared to men and younger individuals. The individuals without filled prescription had also used non-pharmaceutical drugs to a larger extent than those with filled prescriptions.

These observations indicate two distinct groups among individuals who die from medicinal opioids. Approximately half of the study population acquired the medication through prescriptions, predominantly older females, possibly with chronic pain diagnoses [10]. Cancer diagnosis among these individuals did not seem to play an important role. The other half obtain opioids from alternative sources, exhibiting characteristics of illicit or non-medical drug use. These “subgroups” require different prophylactic approaches: prescription users through prescribers and pharmacies, and non-prescription users through other preventive measures.

Previous research showed most overdose victims had recent healthcare contact, making this an ideal venue for intervention and thorough follow-up [16]. This could include advice on safer use, suicide prevention, non-pharmacological treatments, guided deprescribing, and inclusion in OAT for addiction.

While patient support play a crucial role in reducing overdose deaths, prescribers are of utmost importance. Key factors to avoid harmful drug use include increased drug knowledge, improved patient communication, and adherence to national guidelines. Training prescribers on overdose risks and drug interactions can lead to safer prescriptions. Enhanced communication skills help prescribers guide patients on correct drug use, side effects, and overdose response. Staying updated on national guidelines ensures consistent and safe practices. In Norway, a campaign on opioid prescription involved training visits with key messages from national guidelines for chronic non-cancer pain [17]. These 20–25-min one-on-one sessions in physicians’ offices led to a decrease in opioid users in Central Norwegian municipalities compared to non-intervention. This approach might be beneficial in other counties, countries, and settings as well.

The high proportion of prescriptions in individuals with detected codeine (67%) and tramadol (58%) might be explained by the fact that these opioids are considered as weak opioids compared to for example oxycodone, and prescribers might assume that they therefore are “safer.” Prescription of these opioids were associated with concomitant benzodiazepine use, which increases the death risk [18]. It is crucial for prescribing doctors to consider this risk and closely monitor patients using these medicines, especially when deprescribing or terminating treatment is not feasible, even for “weak” opioids. Importantly, reasonable treatment is to avoid prescription of several psychoactive drugs at all.

Some opioids showed a low proportion of recent prescriptions. The 27% fentanyl prescription rate may be due to prior medical treatment, as fentanyl is often used in acute settings [19]. This study lacks data from medical records or pre-hospital/hospital care. Fentanyl could also be acquired illicitly, but The National Criminal Investigation Service in Norway (KRIPOS) reports rare seizures, suggesting medical administration without individual prescriptions [20]. Methadone and buprenorphine, used in OAT, also had low recent prescription rates. KRIPOS notes these are seized more often than fentanyl, and in addition, they may be diverted from OAT programs [21, 22]. NorPD lacks data from non-pharmacy OAT sites, indicating possible higher medical use before deaths, warranting further investigation with outpatient clinic data. Several factors may explain the lower proportion of prescriptions observed. Some drugs might have been dispensed more than 2 months before death, a timeframe not covered in the presented data. However, differences between 1 year and 2 months were analyzed, found to be small, thus excluded. Other explanations include drugs obtained from family, friends, on-line, over the counter from other countries, or the illegal market. Non-prescribed use poses a risk for overdose due to lack of medical supervision [23].

This study revealed that the concomitant use of opioids and benzodiazepines immediately prior to death was prevalent. Notably, benzodiazepines were frequently prescribed to individuals who also had prescriptions for the detected opioids. This combination is well-documented to significantly increase the risk of fatal outcomes and should normally be avoided [18]. It is not recommended in Norway. Moreover, the co-use of multiple psychoactive drugs heightens the risk of pharmacodynamic interactions and addiction and may initiate a detrimental cycle of substance use. The mean number of detected drugs in this study was four, also among individuals with a filled prescription. This underscores the necessity for prescribers to exercise caution when prescribing multiple psychoactive drugs.

Finally, preventing overdoses among individuals that use non-prescribed medications require a comprehensive approach. Access to treatment for substance use disorders (SUD), such as OAT, would be a primary strategy. Second, making harm-reduction approaches available to those outside of treatment, but also to those in SUD treatment, is of importance. Distribution of naloxone, an antidote that can reverse opioid overdose, might be beneficial to individuals that use opioids, and their relatives, to have access to naloxone with knowledge how to use it [24, 25]. Another risk reducing approach might be education and awareness in terms of provided information about the risks of psychoactive medication use. This can include training on recognizing the signs of an overdose and how to administer first aid. Further, harm reduction measures such as access to clean use equipment and guidance on safer use can reduce the risk of overdose and other health harms [26]. Close follow-up and support of persons with substance use problems, especially during vulnerable transitions such as after release from prison or detoxification, is also an overdose preventing approach [27]. Finally, monitoring and early intervention in terms of healthcare professionals being vigilant for signs of substance use and be able to offer early intervention and treatment might be useful [28]. A suicide prevention plan aimed towards patients could also be beneficial since patients with drug use issues have an increased suicide risk [29, 30].

### Strengths and limitations

This study is an integration of biological results with national wide registry data, allowing for a comprehensive understanding of the drugs used prior to death. The dataset is extensive, encompassing most Norwegian overdose cases. Additionally, Norway boasts a high autopsy rate for overdose deaths, approximately 90% [1], and the quality of data from the CoDR and the NorPD is robust. The results provide insights in a Norwegian context and may be generalized to countries with similar health care systems and prescription characteristics.

However, there are several limitations to consider. Treatment received at medical institutions (typically without individual prescription) cannot be captured in any registry, making it impossible to assess its impact. This might specifically influence the results for buprenorphine and methadone. Furthermore, while the deceased may have dispensed a drug at a pharmacy, it does not necessarily mean that it was used or used as the doctor prescribed. Some individuals may have obtained drugs through prescriptions as well as from the illicit market. The ingested dosage and administration route is unknown and might also influence the overdose risk.

The simultaneous detection of multiple drugs in most cases also poses a challenge; if we were to focus solely on mono-intoxications, the statistical power of the analysis would be diminished. Consequently, we were unable to differentiate between primary intoxicants and secondary findings among the various drugs detected. Thus, the results should be interpreted with caution.

Another limitation is the prevalence of morphine in overdose deaths. Due to the complexities involved in its toxicological interpretation, a thorough examination of medical morphine use was not feasible. Initial findings indicated that less than 5% of morphine-positive cases had a filled prescription, suggesting that detection of morphine likely most often is a result of heroin use. Furthermore, some of the drugs in the non-pharmaceutical drug-group might come from prescription, which was not investigated.

While we assume that decedents without a prescription acquired the drug from the illicit market, this assumption may not be totally accurate. Additionally, it remains unclear whether users of pharmaceutical opioids without prescriptions typically use heroin but lacked access at the time of overdose, or if they regularly use medical opioids without legal prescriptions.

## Conclusion

The proportion of overdose deaths in Norway caused by pharmaceutical opioids has increased this century. This study showed that a substantial number of deceased individuals had filled prescriptions for the opioids detected in their blood at autopsy, particularly pertaining to commonly prescribed analgesic medicines, underscoring the health care system as a crucial source of these substances. This study supports the critical need for targeted interventions to reduce opioid overdose deaths. By distinguishing between prescription and non-prescription users, tailored strategies can be developed to address the specific needs of each group. Enhanced training for prescribers, adherence to national guidelines, and comprehensive harm-reduction measures are essential components in preventing overdoses. Continued research and data collection, particularly from other health registries and from outpatient clinics, might further inform effective prevention strategies and improve patient outcomes.

## Data Availability

No datasets were generated or analysed during the current study.
